# Comparative Study on the Nutritional Quality of Peanut in Saline and Non-Saline Land

**DOI:** 10.3390/foods13233751

**Published:** 2024-11-23

**Authors:** Yimin Zhang, Yanmi Li, Xiao Ren, Jieying Gao, Yuchen Wang, Dazhou Zhu

**Affiliations:** 1 School of Food and Biological Engineering, Chengdu University, Chengdu 610000, China; zz8978681@163.com (Y.Z.); liyanmi@stu.cdu.edu.cn (Y.L.); 2 Institute of Food and Nutrition Development, Ministry of Agriculture and Rural Affairs, Beijing 100081, China; rxiao1022@163.com (X.R.); gaojieying203@126.com (J.G.); 82101232184@caas.cn (Y.W.)

**Keywords:** saline soil, peanut, nutrient composition, functional constituent

## Abstract

Saline soils, as a special class of soil types, have unique physicochemical properties that have far-reaching effects on crop growth and quality characteristics. In order to better develop saline soils as a reserve resource, it is particularly important to exploit the potential of saline crops. Peanut, as one of the important crops in saline soils, can have different quality characteristics depending on the differences in soil salinity and alkalinity, as well as growing conditions. In this study, we compared the nutritional quality and functional composition of five peanut varieties grown in coastal saline soils, with the same varieties grown in non-saline soils in similar areas. The results showed significant differences (p<0.05) between saline and non-saline peanuts in the contents of ash, zinc, phosphorus, β-VE, Cis-11-eicosatetraenoic acid, palmitoleic acid, linolenic acid, and total antioxidant removal capacity, whereby the former was higher than the latter by 0.12 g/100 g, 4.1 mg/kg, 321 mg/kg, 8.98 μg/g, 0.36%, 0.01%, and 0.01%, respectively, and the total antioxidant capacity was lower than that of the latter by 9.18 μg Trolxo/g of fresh weight. Sodium element and superoxide dismutase (SOD) activity contents were extremely significantly (p<0.01) different in peanuts grown in both land types, where the former was higher than the latter by 261.9 mg/kg and 285 U/g, respectively. Water, fat, protein, calcium, copper, iron, potassium, magnesium, manganese, Vc, α-VE, total VE, VB3, 5-methyl-tetrahydrofolate, 5-formyl-tetrahydrofolate, total phenols, total flavonoids, ABTS free radical scavenging capacity, DPPH free radical scavenging capacity, fatty acids (except for Cis-11-eicosapentaenoic acid, palmitoleic acid, and linolenic acid), phytosterols, and guanines showed no significant differences (p<0.01). To sum up, the origin and soil environment have an effect on the quality of peanuts. These results also provide a scientific basis for the quality assessment of peanuts in saline soil.

## 1. Introduction

China is the world’s largest country in terms of population and agriculture. A huge demand for food coexists with resource conditions and external environmental constraints, and enormous challenges are faced when trying to ensure national food security. China has a huge land area, but relatively little arable land, and many of these areas are unsuitable for farming due to natural factors such as soil salinisation [1]. Soil salinisation is a global resource and ecological problem, and with 6.7 × 106 hm^2^ of saline land available, China has the third largest area of saline land in the world [2,3]. This further exacerbates the pressure on agricultural production. However, saline–alkaline land, as an important reserve arable land resource, has great potential for development and utilisation. In recent years, relying on technological innovation and scientific research, China has developed saline–alkaline land by widely applying technologies such as smart salt drainage in concealed pipes, precise salt control by drip irrigation on ridges, and the breeding of saline–alkaline-tolerant crops [4], which have significantly improved its utilisation efficiency and promoted the sustainable development of agriculture, which not only provides a strong guarantee of food security in China, but also provides ideas for the global sustainable utilisation of saline–alkaline land resources.

Obtaining a good or bad yield and quality are reflected by the cultivation of saline crops. It is widely believed that the complex salt structure of saline soils, which contain high concentrations of salts and alkaline substances, is detrimental to the growth of crops. The high salt environment interferes with the normal physiological functions of the crop, affecting the absorption and transport of water and nutrients, resulting in stunted growth and often lower yields [5,6,7,8]. However, there is also a view that saline soils are rich in minerals, and these minerals, when absorbed by crops, can enhance the nutritional value of the crops and also give them a unique advantage in quality. However, there is a lack of comprehensive and in-depth research on the specific advantages of saline crops. In this paper, we take peanuts as an example to compare and analyse their nutritional and functional components, and explore the unique advantages in the quality of peanuts in saline and alkaline areas.

Peanuts are rich in proteins, fats, vitamins, minerals, oleic acid, linoleic acid, etc. [9,10,11], and the influence of these rich nutrients on their unique nutritional value and flavour has traditionally been of interest to breeders.However, despite its high nutritional properties, peanuts are also often exposed to mycotoxin contamination [12]. There are numerous varieties of peanut, each of which exhibits different planting characteristics and quality representatives [13,14]. Some studies have shown that the nutritional quality of a peanut is mostly influenced by the combination of varietal genetic characteristics, environmental conditions, and cultivation technical measures [15]. Lin et al. [16] carried out a nutritional quality appraisal of peanuts with five different skin colours, and the results showed that the protein content of red-white, four-grain red, and purple kui three-nut peanuts reached the international standard for the secondary use of peanuts. The fat content of all five peanut types was low, and the kernels were small, with a very good taste for fresh consumption, which verified the findings of Misra et al. [17] that all five types of peanuts are suitable for use as a food processing product. Li et al. [18] studied two high-fat peanuts and found that those with high oleic acid content and low linoleic acid content are easier to store; the monounsaturated fatty acid content is higher than polyunsaturated fatty acid content; and that those with good saturated fatty acid content have better nutritional value. Luo et al. [19], in a study based on the south, north and east of Xinjiang, the mainland Shandong, and a number of regions, including Hebei and Jilin, conducted a nutrient analysis on five different varieties of peanut and found that the same peanut varieties planted in different regions presented certain differences in nutrient content. Xinjiang peanuts were superior to the mainland in oil content, protein, sucrose, and other indicators to varying degrees. Zhao et al. [20] showed that straw and mulch cover could increase crude protein, oleic acid, and oil content of black peanut kernels to different degrees.Xu et al. [21] showed that different amendments would significantly affect the growth characteristics and yield of peanut in saline soil. Xue et al. [22] showed that salt stress would reduce crop yield. Quality is affected differently depending on the type of crop, salt tolerance and degree of salt stress. Rehanguli-ABULA et al. [23] showed that salt in saline soils affects the protein content, vitamin content and mineral content of crops. Xu et al. [24] showed that saline cultivation had a great effect on peanut quality, especially on oleic acid content and oil-to-substance ratio. Gao, Xian, and Ci et al. [25,26,27] found that saline land improvement and a high cultivation of peanut varieties help in the improvement of nutritional quality and yield in saline peanuts.

To sum up, a lot of research has been performed in China and abroad on peanut quality and the improvement of peanuts in saline and alkaline areas, and certain results have been achieved. However, there is still a gap in the comparison of peanut quality between peanuts in saline and non-saline areas. In this study, the nutritional and functional components of peanuts from saline peanuts in Dongying, Shandong Province, and peanuts from non-saline crops of the same variety in similar areas were compared and analysed. This study was conducted to explore the unique nutritional quality and functional quality of peanuts in saline and alkaline areas, and to lay the foundation for further research on other important indicators of peanuts in different soil alkalinity.

## 2. Materials and Experimental Methods

### 2.1. Raw Materials

The project samples were collected in October 2023 (Sample information see Table 1). Saline peanut samples were collected from Mao Tuo Village, Lijin County, Dongying City, Shandong Province, while non-saline peanut samples were collected from Yuhua 625 from Pingdu City, Shandong Province; Yuhua 155 from Henan Academy of Agricultural Sciences; Pu hua 75 from Puyang Academy of Agricultural Sciences, Henan Province; Shan hua 31 from Shandong Agricultural University; and Zhonghua 215 from Wuhan, Hubei Province Oil Crops Research Institute. The harvested crop was dried and shelled after the manual removal of impurities to obtain clean peanut pods. The peanut kernels retaining the red coat were dried in a constant temperature drying oven until the moisture was constant, and then the dried peanut kernels were ground into a fine powder using an electronic grinder, kept in a refrigerator at 4 °C for spare use, and stored until use.

**Figure 1:**
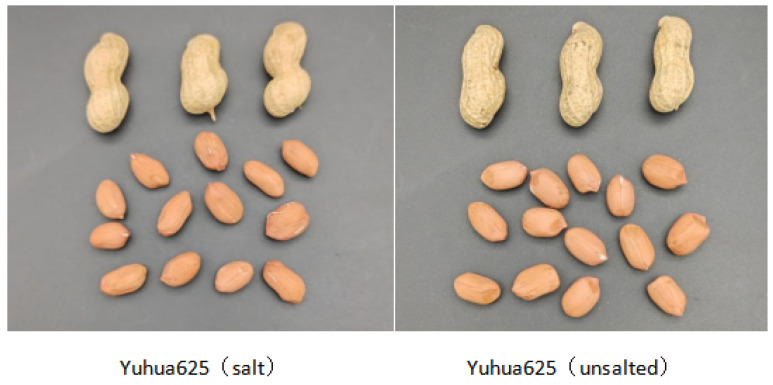
Saline and non-saline peanut phenotypes.

As can be seen from Figure 1, there is no significant difference in appearance between saline and non-saline peanuts of the same variety, and other varieties are not shown here.

### 2.2. Instrumentation and Chemicals

The following were used in this study: an ultrapure water meter (GWA-UN, Beijing Pudian General Instrument Co., Ltd., Beijing, China); analytical balance (AL204-IC, METTLER TOLEDO INSTRUMENTS Co., Ltd., Hongkong); a digestion furnace (XTG5081, Haineng, Rizhao, China); centrifuge (H1750R, Changsha Xiangyi Centrifuge Instrument Co., Ltd., Changsha, China); moisture tester (MA37-1CN, Sartorius Scientific Instruments (Beijing) Co., Beijing, China); Automatic Kjeldahl Nitrogen Determination (K9860, Hainergy, Beijing, China); a microwave disintegrator (MARS 6, CES, Charlotte, NC, USA); an inductively coupled plasma mass spectrometer (Plasma Quant 9100 Agilent, Santa Clara, CA, USA); a high-performance liquid chromatograph (Shimadzu 20AT, Shimadzu Corporation, Kyoto, Japan); a UV-Vis spectrophotometer (ASV11D-H Asia Speedwang (Shanghai) Trading Co., Shanghai, China); and a gas chromatography mass spectrometer (GCMS 2400SQ T Perkin Elmer Instruments Ltd., Hongkong).

α-VE, γ-VE, and δ-VE standards were obtained from Sigma Company, St. Louis, MO, USA; methanol (chromatographic purity) and acetonitrile (chromatographic purity) were from Fisherscientific Company, Waltham, MA, USA; anhydrous ethyl ether (AR), anhydrous sodium sulfate (AR), and ascorbic acid (AR) were obtained from Sinopharm Chemical Reagent Co., Shanghai, China; anhydrous ethanol (AR) and potassium hydroxide (AR) were obtained from Beijing Chemical Reagent Co., Beijing, China; fatty acid methyl ester (standard) was obtained from Sigma-Aldrich (Madrid, Spain); pyrogallic gallic acid (99%) was obtained from Beijing Bailing Wei Technology Co. Beijing, China; and tris(hydroxymethyl)aminomethane (AR), o-hydroxybenzenetriol (AR), disodium ethylenediaminetetraacetate (AR), and n-hexane (AR) were obtained from Sinopharm Chemical Reagent Co.

### 2.3. Determination of Nutrient Content

#### 2.3.1. Determination of Macronutrients

Moisture content was determined using an MA37-1CN Moisture Tester (Sartorius Scientific Instruments (Beijing) Co., Beijing, China). The peanut samples were powdered and homogenised using a high-speed tissue masher. About 1.2 g of powder was spread onto a metal tray of the moisture meter for determination.

Ash determination was performed with reference to GB 5009.4-2016 [28] (the first method: the determination of total ash in food) as follows: The peanut powder (10 g) was uniformly distributed in the crucible. In an electric hot plate, the powder was then fully charred to no smoke in a high-temperature furnace, at 550 ± 25 °C, and left to burn for 4 h. Then, it was cooled to about 200 °C before being taken out and put into a desiccator to cool for 30 min. When there were no charcoal particles left during burning, this meant complete ashing was attained.

Proteins were determined using a Kjeldahl nitrogen tester (Haineng, K9860) by weighing about 0.5 g of the sample (accurate to 0.0001), placing it inside a digestion tube, and adding 0.4 g of CuSO_4_, 6 g of K_2_SO_4_, and 12 mL of concentrated sulphuric acid. Then, the solution was placed in a digestion oven for digestion until it turned transparent or became clear light green in colour, which was the end point of digestion. A total of 2–3 drops of the indicator were added to the receiving bottle; the distilled sample was titrated with 0.05 mol/L HCl; the end point colour was grey, and the volume V of hydrochloric acid consumed was recorded.

Fat determination with reference to the Soxhlet extraction method for the complete extraction of fat was performed by weighing fully mixed peanut samples to about 3 g (accurate to 0.0001 g), then transferring all of the samples to the filter paper cartridge, which was then put into the Soxhlet extraction cylinder, connected to the receiving bottle, then dried to constant weight by the upper end of the condenser tube of the extractor to add n-hexane to the bottle at a volume of two-thirds of the water heated in the water bath, so that the n-hexane reflux continuously underwent extraction (6∼8 times/h, extraction of 3 h). The receiving bottle was removed; the rotary evaporation of hexane was recovered, and then the sample was dried at 100 ± 5 °C for 1.5 h after weighing. The above operation was repeated until constant weight was reached.

#### 2.3.2. Determination of Mineral Content

Minerals were determined by inductively coupled plasma mass spectrometry (ICP-MS), with the following instrumental conditions: radio-frequency (RF) power: 1500 W; plasma gas flow rate: 15 L/min; carrier gas flow rate: 0.80 L/min; temperature of the atomisation chamber: 2 °C; sample lifting rate: 0.3 r/s; nebulizer: high-salt/concentric nebulizer.

#### 2.3.3. Determination of Vitamin Content

Vitamins were determined by redox titration. The experiment was carried out using oxalic acid as a blank and titrated with a calibrated 2,6 dichloroindophenol sodium solution until the solution took on a pinkish color for 15 s without fading.

VE was determined by Shimadzu high-performance liquid chromatography (SPD-20A). The instrumental conditions were as follows: column: C18 (250 mm, 4.6 mm, 5 μm); column temperature: 30 °C; mobile phase (*v*/*v*): A: 10% water; B: 90% methanol; flow rate: 1.0 mL/min; UV detection wavelength: 294 nm; and injection volume: 20 μL.

VB3 was determined by high-performance liquid chromatography (Shimadzu, AT20). Instrumental conditions: determination column: C18 (particle size 5 μm, 250 mm × 4.6 mm). Column temperature: 25 ± 0.5 °C. Ultraviolet detector: detection wavelength of 261 nm. Mobile phase: 70 mL of methanol, 20 mL of isopropanol, and 1 g of sodium heptanesulfonate, dissolved and mixed with 910 mL of water, and then the pH value was adjusted with perchloric acid (700 μL) to 2.1 ± 0.1, followed by 0.45 μm membrane filtration. Flow rate: 0.7 mL/min. Injection volume: 10 μL.

Folic acid was determined by HPLC-MS/MS (3500 AB). Instrumental conditions: mobile phase A was 0.1% formic acid in water, mobile phase B was 0.1% formic acid in methanol; column oven temperature (oven temp): 40 °C; column: Agilent Zorbax SB-C18 (3.5 μm; 3.0 × 150 mm); injection volume: 10 μL; temperature of the sample inlet tank: 4 °C, and all the operations were protected from light; ionisation mode: positive; acquisition mode: MRM; spray voltage: +5500 V; and curtain gas (N2): 20 L/min.

#### 2.3.4. Determination of Phytosterol Content

The peanut sample (2.0 g) was saponified, extracted, filtered, concentrated and then fixed into a 10 mL volumetric flask for the determination of phytosterols using high-performance liquid chromatography (Shimadzu, AT20).

The instrumental conditions were as follows: chromatographic column: Agilent Extend-C18 column (250 mm); mobile phase: acetonitrile/water (98:2 *v*/*v*); flow rate: 1.0 mL/min; column temperature: 30 °C; detection wavelength: 210 nm; and injection volume: 10 μL.

#### 2.3.5. Determination of Guanine Content

The peanut sample (1.000 g) was extracted in a water bath and then fixed to a 50 mL volumetric flask; 2 mL of the filtrate was aspirated, rotary evaporated to near dryness, and dissolved with 2 mL of potassium dihydrogen phosphate solution. Guanine was determined by high-performance liquid chromatography (Shimadzu, AT20).

The instrumental conditions were as follows: chromatographic column: C18 column mobile phase: methanol and potassium dihydrogen phosphate solution ratio of 1:99, isocratic elution; flow rate: 1.0 mL/min; injection volume: 10 μL; column temperature: 30 °C; and determination wavelength: 254 nm.

#### 2.3.6. Determination of Fatty Acid Content

Fatty acids were determined by GC-MS with the following instrumental conditions: chromatographic reference condition: inlet temperature: 270 °C; programmed temperature increase: initial column temperature 40 °C, hold for 1 min, increase the temperature to 210 °C at 7 °C/min, hold for 5 min, and then increase the temperature to 240 °C at 1.5 °C/min; carrier gas and flow rate: high-purity helium (purity > 99.999%), 1.0 mL/min; the reference conditions for mass spectrometry were ionisation mode: electron bombardment ionisation source (EI); ionisation energy: 70 eV; transmission line temperature: 280 °C; ion source temperature: 230 °C; solvent delay: 5 min; and scanning mode: full scanning mode (SCAN)/selected ion scanning (SIM).

### 2.4. Determination of Functional Components

#### 2.4.1. Determination of Superoxide Dismutase (SOD) Activity and Total Antioxidant Capacity

For the determination of superoxide dismutase (SOD) activity, please refer to GB/T 5009.171-2003 [29] “Determination of Superoxide Dismutase (SOD) Activity in Health Foods”, wherein we followed the o-phenyltriol auto-oxidation rate method. Total antioxidant capacity was determined by the total antioxidant capacity (T-AOC) (FRAP method) kit method (Solarbio).

#### 2.4.2. Determination of ABTS Free Radical Scavenging Capacity and DPPH Free Radical Scavenging Capacity

ABTS free radical scavenging capacity was measured by ABTS free radical scavenging capacity assay kit (Solarbio). DPPH free radical scavenging capacity was measured by DPPH free radical scavenging capacity kit (Nanjing Jianjian Bioengineering Institute).

#### 2.4.3. Determination of Total Phenol Content

The total phenols were selected from the laboratory methods. Total volumes of 2 μg/mL, 4 μg/mL, 6 μg/mL, 8 μg/mL, and 10 μg/mL of gallic acid were configured as the standard sample. The gallic acid was weighed to 0.1 g, dissolved in ultrapure water, fixed in a 100 mL volumetric flask, where 1 mL of the volume of a 10 mL volumetric flask constitutes the preparation of a standard solution of 0.1 μg/mL. Then, 0.2, 0.4, 0.6, 0.8, and 1.0 mL of the standard solution was pipetted into a 10 mL volumetric flask, into which 1 mL of Folin reagent and 3 mL of 15% sodium carbonate solution were added sequentially and the solution was fixed to 10 mL with water, then vortexed and oscillated, and the absorbance was measured at 765 nm after 30 min of exposure to light. A blank control was made. The standard curve was plotted with the concentration of the standard substance as the horizontal coordinate and the peak area as the vertical coordinate, and the regression equation was obtained after linear regression.

#### 2.4.4. Determination of Total Flavonoid Content

The total flavonoids were selected from the laboratory method detailed below:

(1) Accurately weigh 15 mg of rutin; dissolve it with 70% ethanol acidic solution until the volume reaches 100 mL of the volumetric flask. Shake well and spare, that is, the standard solution of rutin. Accurately absorb the standard solutions at 0, 0.5, 1, 2, 3, and 4 mL of a 10 mL colorimeter; add 70% ethanol acidic solution to the 5 mL solution, add 0.3 mL 5% NaNO_2_ for 5 min, add 0.3 mL 10% AlCl_3_ for 6 min, and then add 2 mL 1 mol/L NaOH. Add water to the 10 mL solution, and finally measure the absorbance value at a wavelength of 510 nm and establish the standard curve.
(2) Weigh the sample to 20 g. Prepare a homogeneous slurry by adding an appropriate amount of water and then extract it into a conical flask by ultrasonic extraction with 100 mL of 70% acidic ethanol solution. Extraction time should be 40 min; extraction temperature, 40 °C. After cooling, centrifuge the supernatant (3500 r/min, 20 min) into a 100 mL volumetric flask.
(3) Determine the flavonoid content of the above samples by drawing 1.0 mL of the samples separately according to the standard curve method. The sample without reaction solution should be used as the blank sample.

### 2.5. Statistical Analysis

The experimental data were collated using Excel 2010 and correlation analyses were performed using SPSS 23.0 software. In order to analyse the difference between the peanut content of saline-soil and non-saline-soil peanuts, a paired sample analysis was carried out on the averages of saline-soil and non-saline-soil samples. The sample size for both saline and non-saline peanuts was 30, n < 50, so the sample data were tested for normality using the Shapiro–Wilk (S-W) test. p<0.05 on the saline and non-saline sides did not satisfy the requirement of normal distribution, and a non-parametric test (Wilcoxon signed rank test) was used; otherwise, a paired sample *t*-test was used. p<0.05 indicated a significant difference, and p<0.01 indicated an extremely significant difference.

## 3. Results and Discussion

### 3.1. Comparative Analysis of Macronutrient Content

Macronutrients play a vital role in the human body. They are not only the main source of energy, but are also involved in forming the basic structure and function of the body. In the present study, macronutrients were examined in the same variety of saline and non-saline peanuts (Table 2).

In order to analyse the differences in macronutrient contents between saline-soil and non-saline-soil peanuts, paired samples were analysed for saline-soil and non-saline-soil sample means. As can be seen from Table 3, moisture, ash, and fat met the conditions for normal distribution and were analysed for significance using the paired sample *t*-test; protein did not meet the conditions for normal distribution and was analysed for significance using the Wilcoxon signed rank test.

From Table 3, it can be seen that there was no significant difference (p>0.05) in moisture, protein, and fat content between saline and non-saline peanuts, a finding that is at variance with some of the findings of previous studies [30,31,32], which may be due to the influence of the saline soil environment. Ash content was significantly higher (p<0.05) than that of non-saline peanut, with the former being 1.05 times higher than the latter. This finding is similar to the results of Liang et al. [30] and Huang et al. [33], which showed that different soil types and origin environments significantly affected the uptake of ash content in peanuts. It was further verified that the high-salinity environment specific to saline soils led to changes in the accumulation of inorganic salts in peanuts.

### 3.2. Comparative Analysis of Mineral Element Content

Peanuts contain a comprehensive range of minerals, including potassium, phosphorus and magnesium. Copper, zinc, manganese, and other trace elements in peanuts are essential for maintaining normal physiological functions of the human body. The contents of nine mineral elements (calcium, copper, iron, potassium, magnesium, manganese, sodium, phosphorus, and zinc) in the same variety of saline-soil peanuts and non-saline-soil peanuts were determined (Table 4).

In order to analyse the differences in mineral element contents between peanuts from saline and non-saline areas, paired samples were analysed for the mean values of saline and non-saline samples. As can be seen from Table 5, calcium, copper, iron, potassium, phosphorus, and zinc met the conditions for normal distribution and were analysed for significance using the paired sample *t*-test; sodium, magnesium, and manganese did not meet the conditions for normal distribution and were analysed for significance using the Wilcoxon signed rank test.

From Table 5, it can be seen that concentrations of copper, potassium, magnesium, sodium, phosphorus, and zinc are higher in peanuts grown in saline soil than in those grown in non-saline soil. This may be due to the special environmental conditions (e.g., high salinity, high pH, etc.) in saline soils, promoting the accumulation of these elements. It can also be seen that there is no significant difference (p>0.05) in the content of calcium, copper, iron, potassium, magnesium, and manganese between saline and non-saline peanuts. Zinc and phosphorus element contents differ significantly (p<0.05), with the former being 1.12 and 1.07 times higher than the latter, respectively. The difference in sodium element contents is extremely significant (p<0.01), with the former being 2.94 times higher than the latter. Similar to the results of Dai et al. [34], Jia et al. [35], Zhang et al. [36], and Zheng et al. [37], the absorption of different mineral elements by peanut was affected by different soil types and cultivation conditions. The difference in sodium element is extremely significant and 2.94 times higher than that in non-saline soil. This further proves that the special environmental conditions of saline soils promote the accumulation of elements, especially sodium salts, and therefore, peanuts will absorb more sodium elements when growing in saline soils.

**Figure 2:**
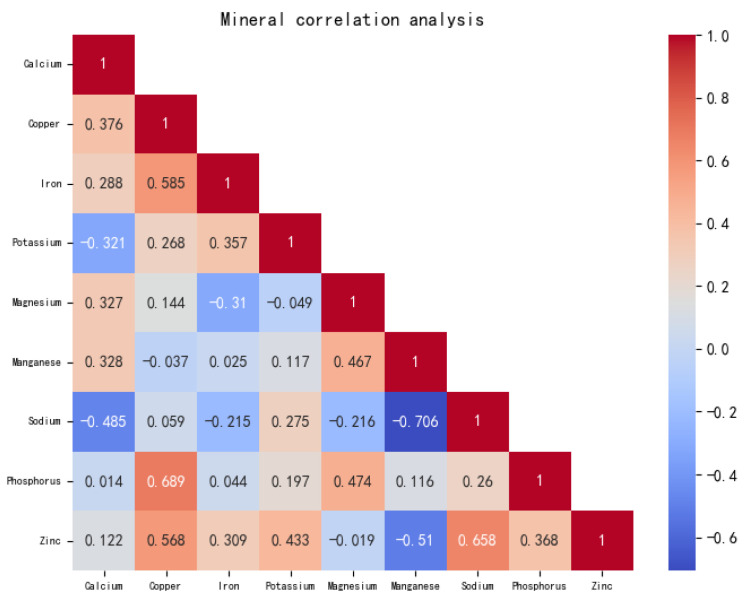
Mineral correlation analysis between saline and non-saline peanuts of the same variety.

According to Figure 2, after a correlation analysis of the contents of nine mineral elements, it was found that minerals in the 10 peanut samples showed four pairs of highly significant positive correlations (*r* <0.01), two pairs of significant positive correlations (r<0.05), one pair of highly significant negative correlations (r<0.01), and two pairs of significant negative correlations (r<0.05). There were highly significant positive correlations between copper and iron, copper and phosphorus, copper and zinc, and sodium and zinc; significant positive correlations between magnesium and manganese, and magnesium and phosphorus; highly significant negative correlations between manganese and sodium; and significant negative correlations between calcium and sodium, and manganese and zinc.

To sum up, the significant differences in the mineral contents of the selected peanuts in this study are the result of the interaction between the minerals in peanuts and the corresponding elements in the soil, and are also related to the uptake and transport of minerals in peanuts.

### 3.3. Comparative Analysis of Vitamin Content

Vitamins are a class of organic substances essential to the human body and play an important role in maintaining human health. In this study, the content of vitamin elements (Vc,β-VE, α-VE, total VE, VB3, 5-formyl-tetrahydrofolate, and 5-methyl-tetrahydrofolate) in peanuts of the same variety of saline and non-saline soil was determined (Table 6).

In order to analyse the differences in vitamin content between peanuts from saline and non-saline areas, paired samples were analysed for the mean values of saline and non-saline samples. As shown in Table 7, total VE, 5-formyl-tetrahydrofolate, and 5-methyl-tetrahydrofolate satisfied the conditions of normal distribution and were analysed for significance using the paired-sample *t*-test; β-VE, α-VE, Vc, and VB3 did not satisfy the conditions of normal distribution, and were analysed for significance using the Wilcoxon signed rank test.

From Table 6, it is clear that the vitamin content of saline-soil peanuts is generally higher than that of non-saline peanuts. This difference may be related to several factors. Table 7 shows that there was no significant difference (p>0.05) between saline and non-saline peanut vitamin elements in Vc, α-VE, total VE, VB3, 5-formyl-tetrahydrofolate, and 5-methyl-tetrahydrofolate contents. β-VE differed significantly (p<0.05), with the former being 2.12 times higher than the latter. Similar to the results of Wang et al. [38], Zhang et al. [39], and Wang et al. [40], the different soil types, cultivation conditions, and environment of origin all affected vitamin uptake in crops. The significant difference in β-VE content further proved that salt stress in saline soil might have stimulated some physiological mechanisms in peanut plants to produce more antioxidants to cope with environmental stresses.

### 3.4. Comparative Analysis of Phytosterol Composition and Content

Phytosterols are widely found in the seeds of oilseed crops and are the main constituents of unsaponifiable oils and fats, mainly consisting of β-sitosterol, rapeseed stanol, and soya stanol. Phytosterols belong to the functional components formed in the combined state of fat and sugar, which cannot be synthesised by themselves in the human body and must be ingested through dietary intake. In this study, the phytosterol content in peanuts from different saline and non-saline sites was determined. The results are shown in Table 8. The average content of β-sitosterol in peanuts from saline sites was higher than that of peanuts from non-saline sites by 0.96 mg/100 g; and leguminol and canola sterols were lower than that of peanuts from non-saline sites.

In order to analyse the differences in phytosterol content between saline and non-saline peanuts, paired samples were analysed for saline and non-saline sample means. As can be seen from Table 9, β-sitosterol, soy sterols, and canola sterols did not satisfy the conditions for normal distribution and were analysed for significance using the Wilcoxon signed rank test.

From Table 9, it is clear that there was no significant difference (p>0.05) in the phytosterol content of peanuts between saline and non-saline sites. There is a discrepancy with the results of Grosso et al. [41], He et al. [42], and Bajguz et al. [43], and it is possible that there is a different variability in peanut’s response to the counter-environment, and this variability may be related to its own physiological mechanisms and adaptive strategies, and may also be affected by peanut varieties. Table 8 shows that both saline and non-saline peanut varieties contain β-sitosterol, soya stanol, canola stanol, three kinds of phytosterols, and all kinds of phytosterols in peanuts in the content order of β-sitosterol > canola stanol > soya stanol. β-sitosterol accounted for the largest proportion of the results, similar to the results of the research of Zhang et al. [39] and Peng et al. [44]. In addition to β-sitosterol and canola sterols, peanuts also contain soya sterols, as detected in this experiment, which is consistent with the findings of Zhang et al. [39] and Feng et al. [45].

### 3.5. Comparative Analysis of Guanine Content

The direct effect of guanine on peanut is limited, but it plays an important role in genetic information storage and signalling as one of the important components in peanut cells. Guanine content was determined in peanuts from different saline and non-saline sites (Table 10).

In order to analyse the differences in phytosterol content between saline and non-saline peanuts, paired samples were analysed for saline and non-saline sample means. As shown in Table 11, guanine did not satisfy the normal distribution condition and was analysed for significance using the Wilcoxon signed rank test.

From Table 11, there was no significant difference (p>0.05) between saline and non-saline peanut guanine content. From Table 10, it can be seen that in addition to Pu Hua 75, the rest of the varieties of peanut saline guanine content is higher than those from non-saline land, Qu et al. [46] and Xu et al. [47] showed that peanut under salt stress physiological and biochemical indexes will produce certain changes. These changes may indirectly reflect the potential impact of saline stress on the DNA (including guanine, including the bases). The fact that the guanine content of Pu Hua 75 in saline soil was lower than that in non-saline soil suggests that there are differences in the response of peanut varieties to saline stress, and that Pu Hua 75 may have unique physiological mechanisms or genetic characteristics that allow it to better adapt to or mitigate the potentially detrimental effects of saline environments on the structure of DNA.

### 3.6. Analysis of Fatty Acid Composition and Relative Content of Peanuts

Fatty acid composition and relative content are closely related to the quality, processing, and storage performance of peanuts. Gas chromatography was used to analyse and determine the fatty acid composition of the same variety of saline peanuts and non-saline peanuts, and the results of the fatty acid composition and relative fatty acid content of the saline and non-saline peanuts are shown in Table 12. As can be seen in Table 12, except for Yuhua No. 155 (non-saline), which did not contain cis,cis-11,14-eicosadienoic acid, the other nine samples contained fifteen kinds of fatty acids, of which there were eight kinds of saturated fatty acids and seven kinds of unsaturated fatty acids. The other 9 samples contained 15 fatty acids, including 8 saturated fatty acids and 7 unsaturated fatty acids. Oleic and linoleic acids are the major fatty acids in peanuts, and the combined percentage of oleic and linoleic acids in both saline and non-saline peanuts was about 80% of the total fatty acids in peanuts, which is consistent with previous studies [48,49,50,51].

As can be seen from Table 13, from the analysis of fatty acid composition, the variation in saturated fatty acid content of peanuts in five kinds of saline and non-saline land ranged from 18.61% to 23.95%, and the average content of saline and non-saline land was 21.58% and 21.38%. The variation in unsaturated fatty acid content ranged from 75.26% to 81.39%, and the average content of saline and non-saline land was 78.42% and 78.22%. Monounsaturated fatty acid content varied from 20.19% to 71.31%, and the average content of saline and non-saline land was 59.43% and 60.02%. Polyunsaturated fatty acid content varied from 4.55% to 61.20%, and the average content of saline and non-saline land was 18.99% and 18.19%. The results of the study through comparative analysis showed that the unsaturated fatty acid content of peanut in saline and alkaline land is higher than the total fatty acid content. It shows that peanuts from saline and alkaline land have better use values.

In order to analyse the differences in fatty acid content between saline and non-saline peanuts, paired samples were analysed for the mean values of saline and non-saline samples. As shown in Table 14, myristic acid, palmitic acid, stearic acid, cis-11-eicosatetraenoic acid, cis,cis-11,14-eicosadienoic acid, erucic acid, eicosatetraenoic acid, and xylocarboxylic acid fulfilled the conditions of normal distribution, and were analysed for significance using a paired-sample *t*-test. Palmitoleic acid, heptadecanic acid, oleic acid, linoleic acid, linolenic acid, arachidic acid, and behenic acid did not satisfy the conditions of normal distribution, and were analysed for significance using the Wilcoxon signed rank test for significance analysis.

As shown in Table 14, there was no significant difference (p>0.05) between saline and non-saline peanuts in terms of fatty acid composition other than cis-11-eicosatetraenoic acid, palmitoleic acid, and linolenic acid. The differences were significant (p<0.05) for cis-11-eicosatetraenoic acid, palmitoleic acid, and linolenic acid, with the former being higher than the latter by 0.36%, 0.01%, and 0.01%, respectively, which is similar to the findings of Shi et al. [52] and Hu et al. [53], that salt stress in saline and alkaline areas affects some of the fatty acid fractions of peanuts.

### 3.7. Analysis of the Relative Content of Functional Components of Peanuts

Phenols and flavonoids are products of plant secondary metabolism, and flavonoids are involved in processes such as plant growth and development and stress response. Free radicals are intermediate metabolites of various biochemical reactions in the body, and their abnormal accumulation can cause oxidative damage to the organism. Phenolic compounds can scavenge free radicals and antioxidant capacity is a compound that can neutralise free radicals, further reducing oxidative stress and protecting biomolecules from potential damage. SOD, as a widespread metalloenzyme in living organisms, is a purely natural type of biologically active substance with no toxic side-effects in humans and animals. In the present study, the relative content of antioxidant functional components in saline and non-saline peanuts was determined (Table 15).

In order to analyse the differences in fatty acid content between saline and non-saline peanuts, paired samples were analysed for the mean values of saline and non-saline samples. As shown in Table 16, total phenols, total flavonoids, total antioxidant capacity, and superoxide dismutase (SOD) activity satisfied the conditions of normal distribution and were analysed for significance using paired-sample *t*-test, while DPPH radical scavenging capacity and ABTS radical scavenging capacity did not satisfy the conditions of normal distribution and were analysed for significance using Wilcoxon signed rank test.

As shown in Table 16, there was no significant difference (p>0.05) between saline and non-saline peanuts in terms of functional components other than total antioxidant capacity and superoxide dismutase (SOD) activity. The difference in total antioxidant exclusion capacity was significant (p<0.05), and the difference in superoxide dismutase (SOD) activity was highly significant (p<0.01), with the former being 1.28 times higher than the latter, similar to the findings of Li et al. [54], Zhang et al. [55], and Xu et al. [56] that salt stress in saline and alkaline environments affects some of the functional constituent components of peanuts. Saline-soil peanuts responded more strongly to the stress of the growing environment, and their adaptability to the external environment was significantly better than that of non-saline-soil peanuts.

**Figure 3:**
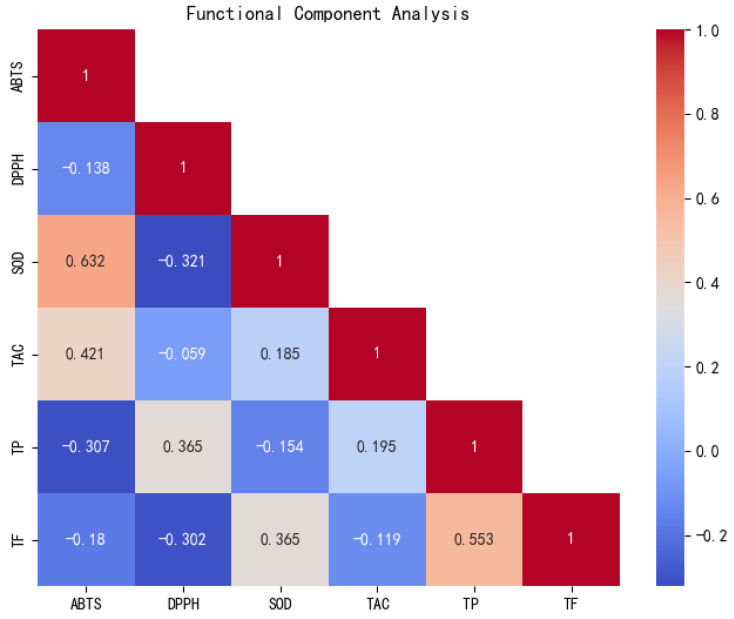
Correlation analysis of functional components of peanut in saline and non-saline soil (TAC: total antioxidant capacity; TP: total phenol; TF: total flavonoids).

According to Figure 3, after a correlation analysis of the contents of the six functional components, it was found that the functional components of the 10 peanut samples showed a highly significant positive correlation (0.01) and significant positive correlation (r < 0.05). ABTS free radical scavenging capacity and SOD activity showed highly significant positive correlation; total phenols and total flavonoids showed a significant positive correlation.

## 4. Conclusions

Fat, protein, vitamins, minerals, and fatty acids in peanuts are important indicators of the intrinsic quality of peanuts. The results of this study showed that the ash content in peanuts from saline soils was significantly (p<0.05) affected by the high-salinity environment unique to saline soils. The high and low ash contents were closely related to the mineral content in the soil of origin and other factors. The significant (p<0.05) difference in zinc and phosphorus contents and the extremely significant (p<0.01) difference in sodium content further indicate that saline soils are rich in minerals. Significant (p<0.05) differences in β-VE, cis-11-eicosatetraenoic acid, palmitoleic acid, linolenic acid, and total antioxidant capacity as well as highly significant (p<0.01) differences in superoxide dismutase (SOD) activity are observed in peanuts from saline and non-saline environments. This indicates that salt stress in saline environments may stimulate some physiological mechanisms in peanut plants and affect some elements’ absorption in peanut; the specific mechanism of the effect needs to be further investigated. It also indicates that the origin and soil environment are very important for the formation of peanut quality. These results provide an important theoretical basis for mining the unique nutritional quality of peanut and its functional quality in saline soil.However, there are limitations in this study, such as the fact that the saline peanut samples were obtained from a specific region, which may limit the applicability of the results to a wide range of quality characteristics that are not fully representative of all saline peanuts. To overcome this limitation, in future studies, the sample will be further expanded to cover more saline peanut samples from different regions. Meanwhile, the potential effects of long-term consumption of saline peanuts on human health will be further investigated to provide a more comprehensive and scientific basis for the development and utilisation of saline peanuts.

## Figures and Tables

**Table 1:** Sample information sheet.

Assortment	Land Type	Sample Site	Salinity–Alkalinity
Yuhua 625	saline soil	Maotuo Village, Lijin County, Dongying City, Shandong Province	0.3% 0.5%
	non-saline soil	Pingdu City, Shandong Province	0
Yuhua 155	saline soil	Maotuo Village, Lijin County, Dongying City, Shandong Province	0.3% 0.5%
	non-saline soil	Henan Academy of Agricultural Sciences	0
Puhua 75	saline soil	Maotuo Village, Lijin County, Dongying City, Shandong Province	0.3% 0.5%
	non-saline soil	Henan Puyang Academy of Agricultural Sciences	0
Shanhua 155	saline soil	Maotuo Village, Lijin County, Dongying City, Shandong Province	0.3% 0.5%
	non-saline soil	Shandong Agricultural University	0
Zhonghua 155	saline soil	Maotuo Village, Lijin County, Dongying City, Shandong Province	0.3% 0.5%
	non-saline soil	Wuhan Oil Institute	0

**Table 2:** Macronutrients in saline-soil and non-saline-soil peanuts.

Assortment	Land Type	Water Content	Ash Content	Protein Content	Fat Content
Yuhua 625	saline soil	3.13	2.65	23.90 ± 0.06	42.12 ± 0.57
	non-saline soil	3.02	2.56	24.41 ± 0.16	43.22 ± 1.70
Yuhua 155	saline soil	2.54	2.69	23.42 ± 0.01	47.18 ± 3.13
	non-saline soil	3.27	2.65	21.44 ± 0.18	49.53 ± 2.09
Puhua 75	saline soil	2.82	2.69	23.33 ± 0.08	44.01 ± 4.79
	non-saline soil	2.58	2.67	20.09 ± 0.11	45.60 ± 4.24
Shanhua 31	saline soil	2.57	2.59	19.15±0.06	50.68 ± 1.65
	non-saline soil	3.63	2.39	24.82 ± 0.19	46.02 ± 1.58
Zhonghua 215	saline soil	1.98	2.72	20.03 ± 0.11	57.85 ± 2.95
	non-saline soil	2.33	2.50	24.75 ± 0.09	55.60 ± 0.91
saline soil		2.60 ± 0.42	2.67 ± 0.05	21.97 ± 2.20	48.37 ± 6.22
non-saline soil		2.96 ± 0.52	2.55 ± 0.11	23.10 ± 2.19	47.99 ± 4.81

Water content unit: %; others’ unit: g/100 g.

**Table 3:** Wilcoxon signed rank, normality test and paired sample *t*-test results for macronutrient content of peanuts in saline and non-saline soils.

	Saline Normality	Non-Saline Normality	Average Value	Standard Deviation	t	*p*	*z*
Water	0.544	0.461	−0.358	0.522	−0.217	0.58	-
Ash	0.088	0.361	0.108	0.126	2.706	0.036 *	-
Fat	0.736	0.753	0.446	3.153	0.447	0.665	-
Protein	0.005 *	0.016 *	-	-	-	0.114	−1.58 ^b^

* denotes p<0.05; ^b^: Based on the negative rank.

**Table 4:** Mineral composition and content of saline and non-saline peanuts.

Mineral	Land Type	Yuhua 625	Yuhua 155	Puhua 75	Shanhua 31	Zhonghua 215	Average Value
	saline soil	640.1 ± 5.30	596.6 ± 0.99	910.0 ± 2.33	801.1 ± 0.85	820.6 ± 3.89	760.7 ± 136
Calcium	non-saline soil	736.2 ± 0.49	555.7 ± 3.89	732.6 ± 2.12	855.8 ± 2.90	1253 ± 16.9	826.7 ± 261
	saline soil	9.592 ± 0.02	13.36 ± 0.14	11.32 ± 0.06	11.17 ± 0.06	13.43 ± 0.13	11.77 ± 1.63
Copper	non-saline soil	7.633 ± 0.08	13.15 ± 0.29	9.622 ± 0.07	10.23 ± 0.05	16.28 ± 0.04	11.38 ± 3.38
	saline soil	24.36 ± 0.40	23.48 ± 0.37	20.48 ± 0.07	18.67 ± 0.04	22.80 ± 0.09	22.13 ± 2.44
Iron	non-saline soil	20.33 ± 0.23	25.35 ± 0.26	24.39 ± 0.06	23.67 ± 0.05	28.86 ± 0.13	24.52 ± 3.07
	saline soil	8735 ± 21.2	8889 ± 75.7	7528 ± 77.1	7943 ± 1.41	9319 ± 91.9	8482 ± 729
Potassium	non-saline soil	7784 ± 10.6	8323 ± 112	9100 ± 41.0	7543 ± 4.95	8296 ± 26.2	8209 ± 599
	saline soil	2144 ± 19.1	2161 ± 1.41	2178 ± 6.36	2535 ± 7.07	2527 ± 7.78	2309 ± 203
Magnesium	non-saline soil	2275 ± 2.12	2233 ± 29.7	2207 ± 4.24	2398 ± 7.07	2300 ± 3.54	2282 ± 73.9
	saline soil	9.44 ± 0.08	9.90 ± 0.07	11.64 ± 0.24	10.81 ± 0.01	22.80 ± 0.10	12.92 ± 5.59
Manganese	non-saline soil	21.21 ± 0.11	12.96 ± 0.01	15.16 ± 0.05	15.72 ± 0.09	16.29 ± 0.06	16.27 ± 3.04
	saline soil	530.2 ± 8.70	523.9 ± 1.63	288.4 ± 2.97	401.7 ± 5.09	239.9 ± 3.32	396.8 ± 132
Sodium	non-saline soil	96.53 ± 0.05	196.2 ± 0.14	130.9 ± 1.20	117.7 ± 0.21	133.3 ± 0.14	134.9 ± 37
	saline soil	4513 ± 26.9	4911 ± 31.9	4575 ± 14.9	4949 ± 19.1	5380 ± 14.1	4865 ± 347
Phosphorus	non-saline soil	4403 ± 3.54	5076 ± 40.3	3918 ± 13.4	4435 ± 1.41	4891 ± 3.54	4544 ± 455
	saline soil	39.18 ± 0.45	37.33 ± 0.42	36.07 ± 0.17	36.67 ± 0.06	37.02 ± 0.16	37.51 ± 0.97
Zinc	non-saline soil	27.86 ± 0.25	33.74 ± 0.44	34.40 ± 0.01	33.30 ± 0.16	37.77 ± 0.23	33.41 ± 3.57

Unit (mg/kg).

**Table 5:** Wilcoxon signed rank, normality test and paired sample *t*-test results for mineral content of peanuts in saline and non-saline soils.

	Saline Normality	Non-Saline Normality	Average Value	Standard Deviation	t	*p*	*z*
Calcium	0.116	0.060	−98.98	205.20012	−1.525	0.162	−
Copper	0.065	0.196	−0.387	1.83066	0.667	0.521	−
Iron	0.180	0.363	−2.560	3.78072	−2.141	0.061	-
Potassium	0.263	0.219	273.800	1003.38912	0.863	0.411	-
Phosphorus	0.112	0.209	320.500	320.89952	3.158	0.012 *	-
Zinc	0.151	0.103	3.840	4.28053	2.837	0.020 *	-
Sodium	0.046 *	0.025 *	-	-	-	0.005 **	−2.803 ^b^
Magnesium	0.001 *	0.168	-	-	-	0.646	−0.459 ^b^
Manganese	0.000 *	0.057	-	-	-	0.203	−1.274 ^b^

* denotes p<0.05; ** denotes p<0.01; ^b^: Based on the negative rank.

**Table 6:** Vitamin composition and content of peanuts in saline and non-saline soil.

Assortment	Land Type	Vc	β-VE	α-VE	total-VE	VB3	5-formyl-tetrahydrofo Late	5-methyl-tetrahydrofo Late
Yuhua 625	saline soil	8.13 ± 0.06	11.01 ± 1.01	5.30 ± 0.10	16.31	5.48 ± 0.01	4.98 ± 0.32	3.01 ± 0.18
	non-saline soil	7.63 ± 0.00	11.34 ± 0.01	8.64 ± 1.12	19.98	4.98 ± 0.05	4.00 ± 0.64	2.53 ± 0.30
Yuhua 155	saline soil	8.66 ± 0.06	6.89 ± 0.96	5.99 ± 0.33	12.88	4.20 ± 0.07	3.52 ± 0.90	1.71 ± 0.05
	non-saline soil	9.43 ± 0.11	5.80 ± 0.29	6.09 ± 0.13	11.88	4.30 ± 0.01	3.62 ± 0.83	2.44 ± 0.00
Puhua 75	saline soil	9.00 ± 0.06	13.46 ± 1.32	10.11 ± 0.26	23.57	4.20 ± 0.01	3.26 ± 0.91	3.58 ± 1.37
	non-saline soil	7.29 ± 0.06	4.90 ± 0.31	2.72 ± 0.14	7.62	6.89 ± 0.04	3.50 ± 0.31	2.11 ± 0.20
Shanhua 31	saline soil	6.50 ± 0.00	28.01 ± 0.58	9.61 ± 1.10	37.61	4.73 ± 0.04	4.27 ± 0.51	2.57 ± 0.25
	non-saline soil	6.17 ± 0.06	11.34 ± 0.03	6.50 ± 0.65	17.84	3.68 ± 0.08	5.05 ± 0.41	1.86 ± 0.20
Shanhua 215	saline soil	6.69 ± 0.06	25.48 ± 0.37	10.28 ± 0.42	35.76	5.33 ± 0.09	3.52 ± 0.10	3.25 ± 0.03
	non-saline soil	6.96 ± 0.00	6.57 ± 0.57	5.08 ± 0.61	11.64	3.38 ± 0.01	3.02 ± 0.11	1.38 ± 0.25
saline soil		7.79 ± 1.08	16.97 ± 8.77	8.26 ± 2.31	25.23 ± 11.17	4.79 ± 0.57	3.91 ± 0.81	2.82 ± 0.83
non-saline soil		7.49 ± 1.14	7.99 ± 2.95	5.80 ± 2.09	13.79 ± 5.03	4.65 ± 1.32	3.84 ± 0.82	2.06 ± 0.47

Vc, VB3 unit: mg/100 g; others’ unit: μg/g.

**Table 7:** Wilcoxon signed rank, normality test and paired sample *t*-test results for vitamin content of peanuts in saline and non-saline soils.

	Saline Normality	Non-Saline Normality	Average Value	Standard Deviation	t	*p*	*z*
total-VE	0.382	0.619	11.434	12.12247	2.109	0.103	-
5-methyl-tetrahydrofolate	0.487	0.853	0.4715	0.07492	1.99	0.078	-
5-formyl-tetrahydrofolate	0.943	0.357	0.0745	0.68501	0.344	0.739	-
α-VE	0.014 *	0.764	-	-	-	0.93	−1.682 ^b^
β-VE	0.074	0.008 *	-	-	-	0.013 *	−2.497 ^b^
Vc	0.033 *	0.110	-	-	-	0.386	-0.867 ^b^
VB3	0.041 *	0.045 *	-	-	-	0.575	−0.567 ^b^

* denotes p<0.05; ^b^: Based on the negative rank.

**Table 8:** Phytosterol composition and content of peanuts from saline and non-saline sites.

Assortment	Land Type	β-Sitosterol	Stigmastero	Campesterol
Yuhua 625	saline soil	31.08 ± 1.76	5.91 ± 0.35	9.54 ± 0.79
	non-saline soil	27.14 ± 0.97	4.74 ± 0.11	15.38 ± 1.44
Yuhua 155	saline soil	32.61 ± 1.43	3.38 ± 0.03	14.02 ± 0.62
	non-saline soil	36.79 ± 1.59	6.03 ± 0.49	13.59 ± 0.71
Puhua 75	saline soil	30.45 ± 2.18	5.34 ± 0.24	12.87 ± 0.02
	non-saline soil	17.91 ± 0.28	4.23 ± 0.05	41.23 ± 0.36
Shanhua 31	saline soil	42.74 ± 1.98	5.31 ± 0.18	12.60 ± 0.77
	non-saline soil	27.26 ± 1.22	7.30 ± 0.64	22.65 ± 0.53
Shanhua 215	saline soil	52.01 ± 5.44	18.39 ± 0.79	42.42 ± 0.47
	non-saline soil	75.03 ± 0.62	20.35 ± 0.64	40.62 ± 0.01
saline soil		37.78 ± 9.12	7.67 ± 5.73	18.29 ± 12.82
non-saline soil		36.82 ± 21.11	8.53 ± 6.34	26.69 ± 12.67

Unit (mg/100 g).

**Table 9:** Wilcoxon signed rank, normality test for phytosterol content in peanuts from saline and non-saline sites.

	β-Sitosterol	Stigmasterol	Campesterol
Saline normality	0.078	0.000 *	0.000 *
Non-saline normality	0.005 *	0.000 *	0.011 *
*z*	−0.357 ^b^	−1.785 ^b^	−1.1886 ^b^
*p*	0.721	0.074	0.059

* denotes p<0.05; ^b^: Based on the negative rank.

**Table 10:** Guanine content of saline and non-saline land.

Assortment	Land Type	Guanine
Yuhua 625	saline soil	0.24
	non-saline soil	0.13
Yuhua 155	saline soil	0.25
	non-saline soil	0.12
Puhua 75	saline soil	0.18
	non-saline soil	0.25
Shanhua 155	saline soil	0.10
	non-saline soil	0.04
Zhonghua 155	saline soil	0.22
	non-saline soil	0.20
saline soil		0.2
non-saline soil		0.15

Unit (mg/g).

**Table 11:** Test of normality of peanut guanine content in saline and non-saline soil and paired sample *t*-test results.

	Saline Normality	Non-Saline Normality	Average Value	Standard Deviation	t	*p*
Guanine	0.064	0.324	0.047	0.075	1.99	0.078

**Table 12:** Analysis of fat composition and relative content of peanuts in saline and non-saline soils.

Chemical Compound	Land Type	Yuhua 625	Yuhua 155	Puhua 75	Shanhua 31	Zhonghua 215	Average Value
Myristic acid	saline soil	0.02%	0.02%	0.02%	0.02%	0.02%	0.02%
	non-saline soil	0.02%	0.01%	0.02%	0.02%	0.02%	0.018%
Palmitic acid	saline soil	9.80%	9.61%	9.52%	10.86%	9.43%	9.84%
	non-saline soil	8.96%	8.90%	9.91%	11.36%	9.63%	9.75%
Palmitoleic acid	saline soil	0.10%	0.10%	0.10%	0.04%	0.16%	0.10%
	non-saline soil	0.07%	0.08%	0.08%	0.04%	0.17%	0.09%
Seventeen carbonates	saline soil	0.10%	0.11%	0.17%	0.04%	0.07%	0.10%
	non-saline soil	0.11%	0.12%	0.10%	0.05%	0.32%	0.14%
Stearate	saline soil	4.60%	5.94%	6.87%	3.58%	6.13%	5.42%
	non-saline soil	5.21%	6.40%	6.00%	4.08%	8.61%	6.06%
Oleic acid	saline soil	67.43%	70.06%	68.25%	20.15%	70.36%	59.25%
	non-saline soil	70.43%	71.22%	66.50%	20.69%	70.56%	59.88%
Linoleic acid	saline soil	9.18%	6.43%	5.83%	60.55%	5.19%	17.44%
	non-saline soil	7.54%	5.51%	9.78%	58.20%	3.56%	16.92%
Linolenic acid	saline soil	0.07%	0.44%	0.04%	0.05%	0.04%	0.05%
	non-saline soil	0.05%	0.04%	0.04%	0.04%	0.03%	0.04%
Arachidonic acid	saline soil	1.52%	2.19%	2.21%	1.15%	2.24%	1.86%
	non-saline soil	1.61%	2.41%	2.15%	1.47%	0.72%	1.67%
Cis-11-Eicosate traenoic acid	saline soil	2.14%	1.41%	1.70%	0.57%	1.58%	1.48%
	non-saline soil	1.70%	1.32%	1.50%	0.72%	0.86%	1.12%
Cis,cis-11, cis-14- Eicosadienoic acid	saline soil	0.05%	0.00%	0.03%	0.02%	0.01%	0.02%
	non-saline soil	0.02%	ND	0.01%	0.02%	0.01%	0.02%
Wasabiic acid	saline soil	2.86%	2.57%	3.41%	1.98%	2.96%	2.76%
	non-saline soil	2.52%	2.48%	2.63%	2.26%	3.46%	2.67%
Erucic acid	saline soil	0.15%	0.07%	0.10%	0.01%	0.08%	0.08%
	non-saline soil	0.10%	0.07%	0.06%	0.01%	0.08%	0.06%
23-carbonate	saline soil	0.04%	0.03%	0.04%	0.02%	0.03%	0.03%
	non-saline soil	0.04%	0.03%	0.04%	0.02%	0.05%	0.04%
Lignocaine	saline soil	1.94%	1.41%	1.72%	0.95%	1.70%	1.54%
	non-saline soil	1.63%	1.41%	1.17%	1.02%	1.94%	1.43%
Oil substitution	saline soil	7.34%	10.90%	6.80%	0.33%	19.83%	8.77%
	non-saline soil	9.34%	12.92%	11.71%	0.36%	13.57%	9.85%

ND: not detected.

**Table 13:** Composition of various fatty acids in saline and non-saline peanuts.

Chemical Compound	Land Type	Yuhua 625	Yuhua 155	Puhua 75	Shanhua 31	Zhonghua 215	Average Value
monounsaturated fatty acid	saline soil	67.68%	70.23%	68.45%	20.19%	70.61%	59.43%
	non-saline soil	70.61%	71.31%	66.65%	20.74%	70.81%	60.02%
polyunsaturated fatty acid	saline soil	11.45%	7.88%	7.60%	61.20%	6.81%	18.99%
	non-saline soil	9.31%	6.87%	11.33%	58.91%	4.55%	18.19%
unsaturated fatty acid	saline soil	79.13%	78.11%	76.05%	81.39%	77.42%	78.42%
	non-saline soil	79.92%	78.24%	77.98%	79.71%	75.26%	78.22%
saturated fatty acid (SFA)	saline soil	20.87%	21.89%	23.95%	18.61%	22.58%	21.58%
	non-saline soil	20.08%	21.76%	22.02%	20.29%	22.74%	21.38%
MUFA/PUFA	saline soil	5.91	8.91	9.01	0.33	10.37	6.91
	non-saline soil	7.58	10.38	5.88	0.35	15.56	7.95

**Table 14:** Wilcoxon signed rank, normality test and paired sample *t*-test results for peanut fatty acid content in saline and non-saline soils.

	Saline Normality	Non-Saline Normality	Average Value	Standard Deviation	t	*p*	*z*
Myristic acid	0.440	0.846	0.0183	0.00484	1.195	0.263	-
Palmitic acid	0.423	0.493	0.4790	0.86518	0.175	0.865	-
Cis-11-Eicosatetraenoic acid	0.214	0.198	0.2642	0.32590	2.564	0.030 *	-
Cis-11,14-Eicosadienoic acid	0.104	0.573	0.0105	0.14988	2.213	0.054	-
Stearic acid	0.186	0.230	−0.6559	1.16368	−1.782	0.108	-
Erucic acid	0.315	0.295	0.1781	0.02802	2.010	0.075	-
23-carbonate	0.115	0.320	−0.0028	0.00740	−1.182	0.267	-
Lignocaine	0.161	0.371	0.1121	0.30739	1.153	0.279	-
Palmitoleic acid	0.384	0.043 *	-	-	-	0.037 *	−2.090 ^b^
Seventeen carbonates	0.271	0.003 *	-	-	-	0.203	−1.274 ^b^
Oleic acid	0.000 *	0.000 *	-	-	-	0.114	−1.580 ^b^
Linoleic acid	0.000 *	0.000 *	-	-	-	0.386	−0.866 ^b^
Linolenic acid	0.016 *	0.722	-	-	-	0.022 *	−2.293 ^b^
Arachidonic acid	0.016 *	0.637	-	-	-	0.646	−0.459 ^b^
Wasabiic acid	0.192	0.014 *	-	-	-	0.575	−0.561 ^b^

* denotes p<0.05; ^b^: Based on the negative rank.

**Table 15:** Analysis of the relative content of functional components of peanut in saline and non-saline land.

Assortment	Land Type	Total Phenol	Total Flavonoids	SOD	DPPH	ABTS	Total Antioxidant Capacity
Yuhua 625	saline soil	212.82 ± 1.87	592.31 ± 5.57	1697 ± 139	189.69 ± 6.01	92.28 ± 0.76	37.60 ± 1.54
	non-saline soil	166.70 ± 6.36	501.61 ± 5.43	1288 ± 79.5	192.69 ± 2.67	87.82 ± 6.48	34.47 ± 3.82
Yuhua 155	saline soil	246.59 ± 1.33	743.90 ± 4.45	1779 ± 368	274.47 ± 2.32	94.10 ± 0.06	35.15 ± 3.58
	non-saline soil	264.98 ± 3.37	419.44 ± 0.06	1493 ± 215	271.20 ± 3.56	94.10 ± 0.18	52.97 ± 3.93
Puhua 75	saline soil	152.51 ± 2.05	158.66 ± 2.35	1308 ± 177	272.14 ± 0.74	91.37 ± 1.69	31.58 ± 1.03
	non-saline soil	210.73 ± 6.88	296.85 ± 5.20	1070 ± 109	262.61 ± 5.28	94.67 ± 0.06	56.59 ± 2.57
Shanhua 31	saline soil	183.51 ± 6.92	288.11 ± 4.46	1124 ± 310	280.56 ± 4.90	91.55 ± 1.43	28.70 ± 7.93
	non-saline soil	252.30 ± 0.75	420.88 ± 1.37	660 ± 158	283.36 ± 0.30	92.90 ± 1.40	32.39 ± 9.34
Zhonghua 215	saline soil	267.22 ± 4.86	604.11 ± 6.63	689 ± 33.2	271.61 ± 1.87	51.49 ± 7.17	28.49 ± 9.00
	non-saline soil	228.11 ± 2.03	386.69 ± 0.38	660 ± 35.3	270.87 ± 1.13	59.04 ± 5.01	30.98 ± 4.50
saline soil		212.53 ± 46.36	477.42 ± 243.8	1319 ± 444	257.69 ± 38.18	84.16 ± 18.29	32.30 ± 4.01
non-saline soil		224.56 ± 38.58	405.09 ± 73.87	1034 ± 373	256.12 ± 36.24	85.71 ± 15.15	41.48 ± 12.27

Total phenol, total flavonoid unit mg/100 g; Sod activity unit U/g; DPPH units μmol/g; ABTS unit %; total antioxidant capacity unit μg Trolxo/g fresh weight.

**Table 16:** Wilcoxon signed rank, normality test and paired sample *t*-test results for functional constituent content of peanuts in saline and non-saline soils.

	Saline Normality	Non-Saline Normality	Average Value	Standard Deviation	t	*p*	*z*
Total phenol	0.435	0.342	−12.0320	50.48858	−0.754	0.470	-
Total flavonoids	0.064	0.215	73.3890	195.11871	1.173	0.271	-
Total antioxidant capacity	0.712	0.231	−9.1760	11.12894	−2.607	0.028 *	-
SOD	0.089	0.274	285.3090	273.72506	4.661	0.000 **	-
ABTS	0.000 *	0.000 *	-	-	-	0.386	−0.868 ^b^
DPPH	0.000 *	0.001 *	-	-	-	0.386	−0.866 ^b^

* denotes p<0.05; ** denotes p<0.01; ^b^: Based on the negative rank.

## Data Availability

The original contributions presented in this study are included in the article. Further inquiries can be directed to the corresponding author.
